# Unraveling thalassemia intermedia: Novel insights of a hemoglobin Jax [HBA2:c.44G>C] and deletional α^0^-thalassemia interaction phenotype

**DOI:** 10.1016/j.htct.2025.103739

**Published:** 2025-02-15

**Authors:** Sitthichai Panyasai, Kanokwan Jaiping, Pisuttinee Khantarag, Patcharee Nochod, Surada Satthakarn

**Affiliations:** a School of Allied Health Sciences, University of Phayao, Phayao, Thailand; b Lamphun Hospital, Lamphun, Thailand; c Faculty of Allied Health Sciences, Burapha University, Chonburi, Thailand

**Keywords:** Unstable hemoglobin, Alpha-thalassemia, Hemoglobin H disease, High-performance liquid chromatography, Capillary Electrophoresis

## Abstract

**Objective:**

To elucidate the molecular basis, hematological features, and electrophoretic and chromatographic mobility behavior of an unstable α_2_-globin chain variant, and to describe the diagnostic approach.

**Methods:**

A Thai patient with unexplained chronic anemia and her daughter were investigated. Hematological data were analyzed using a standard automated cell counter. Hemoglobin was analyzed using high-performance liquid chromatography (HPLC) and capillary electrophoresis (CE). Mutational analysis was performed using appropriate polymerase chain reaction (PCR) techniques and direct sequencing. Additionally, α-globin haplotype analysis was conducted. Simple and rapid diagnostic methods were developed.

**Results:**

Hemoglobin analysis in the patient revealed anomalous peaks separated from normal hemoglobin visible using the HPLC technique. These peaks were virtually absent in the daughter. DNA analysis identified a G to C mutation at codon 14 of the α_2_-globin gene responsible for hemoglobin Jax in *trans* to the α^0^-thalassemia gene in the patient. Heterozygosity of this mutation was identified in her daughter. Hematological analysis showed mild thalassemia-like changes in simple heterozygotes and exhibited a hemoglobin H-like phenotype when combined with α^0^-thalassemia. Isopropanol stability testing and bioinformatic software indicated that the variant was unstable and potentially damaging. This mutation was confirmed using allele-specific PCR. Hemoglobin Jax was strongly associated with the haplotype [+ - *S* + - + -].

**Conclusions:**

Hemoglobin Jax, a pathological α-globin variant, is asymptomatic in simple heterozygotes and demonstrates more pronounced clinical effects when associated with deletional α-thalassemia. This knowledge can help develop strategies to prevent hemoglobinopathies in regions of high prevalence. Accurate identification requires DNA level analysis.

## Introduction

Hemoglobinopathies represent the most prevalent monogenic disorders, characterized by anomalies in globin gene expression leading to the synthesis of structurally abnormal globin chains or an insufficient production of globin chains, causing thalassemia.^1^ Often, the discovery of hemoglobin (Hb) variants occurs serendipitously during the course of thalassemia and hemoglobinopathy control and prevention efforts. Advancements in Hb separation techniques, allow for finer distinctions between rarer variants and Hb A or Hb A_2_, which along with improvements in molecular diagnostic tools, have led to the identification of an increasing number of Hb variants. The HbVar database has recorded approximately 1420 human Hb variants to date.^2^ The clinical impact of Hb variants can range from clinically silent to symptomatic expression, contingent upon the specific mutation site. Within the group of pathogenic Hb variants, clinically relevant outcomes arise due to mutations that alter the intersubunit contacts of α1β1 or α1β2. These modifications confer instability upon the α-globin chain, making it prone to denaturation, proteolytic degradation, and membrane binding, triggering hemolysis.^3^^,^^4^ Mutations can disrupt structural elements proximal to the heme group, destabilizing heme interactions and directly affecting oxygen binding.^5^ While many Hb variants exhibit limited clinical significance and are linked to normal red blood cell (RBC) parameters, particularly in heterozygotes, there exists variants that induce mild to severe α^+^-thalassemia phenotypes and serous clinical symptoms. These variants are often associated with deletional α-thalassemia, Hb Constant Spring, and Hb Paksé, which are prevalent in Southeast Asia.^6^^,^^7^ Moreover, rare unstable variants, such as Hb-Quong Sze and Hb-Suan-Dok, which follow analogous mechanisms, contribute to mild to severe Hb H disease within different populations.^7^, ^8^, ^9^, ^10^, ^11^, ^12^, ^13^, ^14^, ^15^

However, a notable challenge arises in diagnosing unstable Hb variants, as they can evade detection due to their asymptomatic or milder phenotypes. Moreover, they might be misdiagnosed since their electrophoretic mobility matches that of normal structurally intact Hb. Employing a combination of methods, such as Hb high-performance liquid chromatography (HPLC), Hb electrophoresis, Heinz body assessment, and tests evaluating heat and isopropanol stability, emerges as a strategy to reveal these elusive variants. While most abnormal Hbs constitute non-pathological variants, a subset of them can cause clinical symptoms associated with hemolytic anemia. Consequently, precise identification and differential diagnosis of these variants is essential. In this study, the clinical and molecular characterization of a rare unstable Hb variant in a Thai patient, who presented with an unexplained moderate anemia, is described.

## Materials and methods

### Subjects and hematological analysis

A Thai woman presented with chronic moderate anemia without any history of organomegaly or transfusion. While investigating the etiology, an aberrant Hb peak was identified on the chromatogram. Consequently, her daughter was recruited to participate in the study. After obtaining written informed consent, venous blood was collected from both participants, with one sample anticoagulated using ethylenediaminetetraacetic acid (EDTA) and a second without anticoagulant. Hematological analyses were performed using a SysmexXN-3000 automated blood cell counter (Sysmex Corporation, Kobe, Japan). Iron profiles (serum iron, serum ferritin, total iron binding capacity, and transferrin saturation) were determined using a Siemens Atellica IM analyzer (SIEMENS-Healthineers AF, Erlangen, Germany) and a Cobase601 analyzer (Roche Diagnostics, Mannheim, Germany). Hb analysis was conducted by capillary electrophoresis (MINICAP Flex Piercing; Sebia, Lisses, France) and two automated cation-exchange HPLC systems: Premier Resolution (Trinity Biotech, Bray, Country Wicklow, Ireland) using the high-resolution mode, and Variant-II (Bio-Rad Laboratories, Hercules, CA, USA) using the β-Thalassemia Short Program. Hb stability was evaluated using isopropanol precipitation. Stroma-free lysates were heated at 37 °C in a buffer containing 17% isopropanol.^16^ The presence of inclusion bodies within RBCs was detected by incubation with 1% brilliant cresyl blue at 37 °C for 3 h.^17^ For microscopic examination, air-dried peripheral blood smears were prepared and 5000 RBCs were examined.

This study was performed in accordance with the Helsinki Declaration and received approval from the Institutional Review Board (IRB) of the University of Phayao, Thailand (Approval number: 1.2/021/65). Written informed consent was obtained from both participants included in the study.

### Molecular characterization

Genomic DNA was extracted from peripheral blood leukocytes using the Blood DNA Extraction Kit (Vivantis Technologies, Selangor, Malaysia). Identification of five common α-thalassemia deletions, namely Southeast Asian (–^SEA^), THAI (–^THAI^), Chiang Rai (–^CR^), 3.7 kb (rightward) (-α^3^^.^^7^), and 4.2 kb (leftward) (-α^4^^.^^2^) was performed using gap-PCR.^18^, ^19^, ^20^ Direct DNA sequencing was performed for non-deletional α-thalassemia mutations previously documented in the Thai population and other mutations. Selective amplification of α_1_- and α_2_-globin genes was performed according to the protocol by Dodé et al.^21^ After amplification, the products were purified using a GF-1 AmbiClean kit (Vivantis Technologies), and direct DNA sequencing was conducted using an ABI PRISM 3130xl automated analyzer (Applied Biosystems, Foster City, California, USA).

### α-globin haplotype analysis

To demonstrate the evolution and origin of Hb Jax, the α-globin gene haplotypes were determined using seven common polymorphisms. This included six restriction fragment length polymorphisms (RFLPs): *Xba*I site of the 5′ ζ2 globin gene, *Bgl*I sites of the inter ζ-globin gene, *Acc*I site of the 3′ ψα2-globin gene, *Rsa*I site of the 5′ α2-globin gene, *Pst*I sites of the 5′ α1- and 5′ θ1 globin gene, and one broadly triallelic inter ζ-globin hypervariable region (HVR). Amplification of the RFLP and inter ζ-globin HVR regions were performed.^22^ Haplotypes were constructed by determining the presence or absence of cleavage per site and consolidating the outcomes into a single pattern.

### Bioinformatics analysis of hemoglobin variant

To enhance our comprehension of the impact of the amino acid substitution within the α-globin chain, structural alterations at the affected positions were analyzed using the Sorting Intolerant from Tolerant (SIFT) web server (https://sift.bii.a-star.edu.sg/) and the network-based HumDiv-trained Polymorphic Phenotype V.2 (PolyPhen-2) (http://genetics.bwh.harvard.edu/pph2/). Furthermore, the tertiary protein structure was modeled using the SWISS-MODEL (https://swissmodel.expasy.org/). The 3D structures of selected mutant proteins were constructed through the PyMOL prediction model (PyMOL Molecular Graphics System, Version 2.5 Schrodinger, LLC; https://pymol.org/2/). The human Hb protein structure was used as a template (PDB code:1BZ1).

### Allele-specific polymerase chain reaction development for Hb Jax confirmation

A novel allele-specific PCR technique was devised to validate and expedite the diagnosis of Hb Jax, characterized by a G to C transition at codon 14 of the α_2_-globin gene. The specific reverse primer SP26 (5′-GTGCGCGCCGACCTTACCCG-3′), located within exon 1 of the α_2_-globin gene, was combined with forward primer C1 (5′-TGGAGGGTGGAGACGTCCTG-3′), positioned 5′ upstream, to generate a 302-bp fragment specific to the α^Jax^ allele. For internal control of the PCR amplification, primers C1 and C3 (5′-CCATTGTTGGCACATTCCGG-3′) were used to produce a 1085-base pair (bp) fragment. The PCR reaction mixture (50 μL) included genomic DNA, primer C1 (60 pmoles), primer SP26 (1.25 pmoles), primer C3 (30 pmoles), dNTPs (200 μM), and *Taq* DNA polymerase (2.0 units; Vivantis Technologies) in a buffer containing Tris–HCl (10 mM; pH 9.1), 50 mM KCl and 0.1% Triton X-100. Amplification was conducted using a thermal cycler (FlexCycler2 Thermal Cyclers; Analytik Jena AG, Germany). After an initial denaturation step at 94 °C for 3 mins, the reaction involved ten cycles of 94 °C for 30 s, 65 °C for 30 s, and 68 °C for 2 min, succeeded by 20 cycles of 94 °C for 30 s, 65 °C for 30 s, and 68 °C for 2 min plus an incremental time of 20 s per cycle. The amplified product was assessed by electrophoresis in a 1.5% agarose gel and visualized under UV light after ethidium bromide staining.

## Results

### Hematological analysis

Table 1 presents the results of the hematological analyses conducted on the participants. The patient exhibited moderate anemia with a marked decrease in mean corpuscular volume (MCV) and mean corpuscular Hb (MCH). Meanwhile, the daughter displayed mild anemia with marginally decreased MCV and MCH. The patient's reticulocyte count was markedly elevated (4.16%), indicative of heightened erythropoiesis. In contrast, the daughter exhibited a reticulocyte count of approximately 1.3%. The iron profile revealed no irregularities in the patient's iron metabolism. However, her daughter displayed marginally reduced ferritin levels, implying diminished iron storage.

**Table 1:** Hematological data, red blood cell indices, and α-globin genotypes of the patient and her daughter carrying Hb Jax.

Parameters	Patient	Daughter	Reference range
RBC count (× 10^12^/L)	5.04	4.70	3.8–5.5
Hb (g/L)	87	113	120–160
Hct (L/L)	32.6	35.3	36.0–46.0
MCV (fL)	64.6	75.1	80.0–97.0
MCH (pg)	17.3	24.0	27.0–31.0
MCHC (g/dL)	26.8	31.9	32.0–36.0
RDW-CV (%)	26.7	16.0	11.0–15.0
Reticulocyte (%)	4.16	1.34	0.2–2.0
Inclusion bodies (%)	Present (<1%)	Absent	Absent
Iron profile			
Ferritin (μg/L)	243.4	5.1	21.8–274.6
Serum iron (μg/dL)	84.3	91.9	70.0–180.0
TIBC (μg/dL)	271.0	350.8	250.0–460.0
Tsat (%)	33.5	27.6	20.0–50.0
α-globin genotype	–^SEA^/α^14G>C^α	α^14G>C^α/αα	αα/αα
α-haplotype	α^14G>C^α : [+ - *S* + - + -]	α^14G>C^α: [+ - *S* + - + -]	–
	–^SEA^: [+ - S 0 0 0 0]	αα: [+ - *S* + - + -]	–

RBC, Red blood cells; Hb, Hemoglobin; Hct, Hematocrit; MCV, Mean corpuscular volume; MCH, Mean corpuscular hemoglobin; RDW-CV, Coefficient of variation of the red cell distribution width; TIBC, Total iron-binding capacity; Tsat, Transferrin saturation; 0 indicates deletion.

### Hb analysis using high-performance liquid chromatography and other techniques

**Figure 1:**
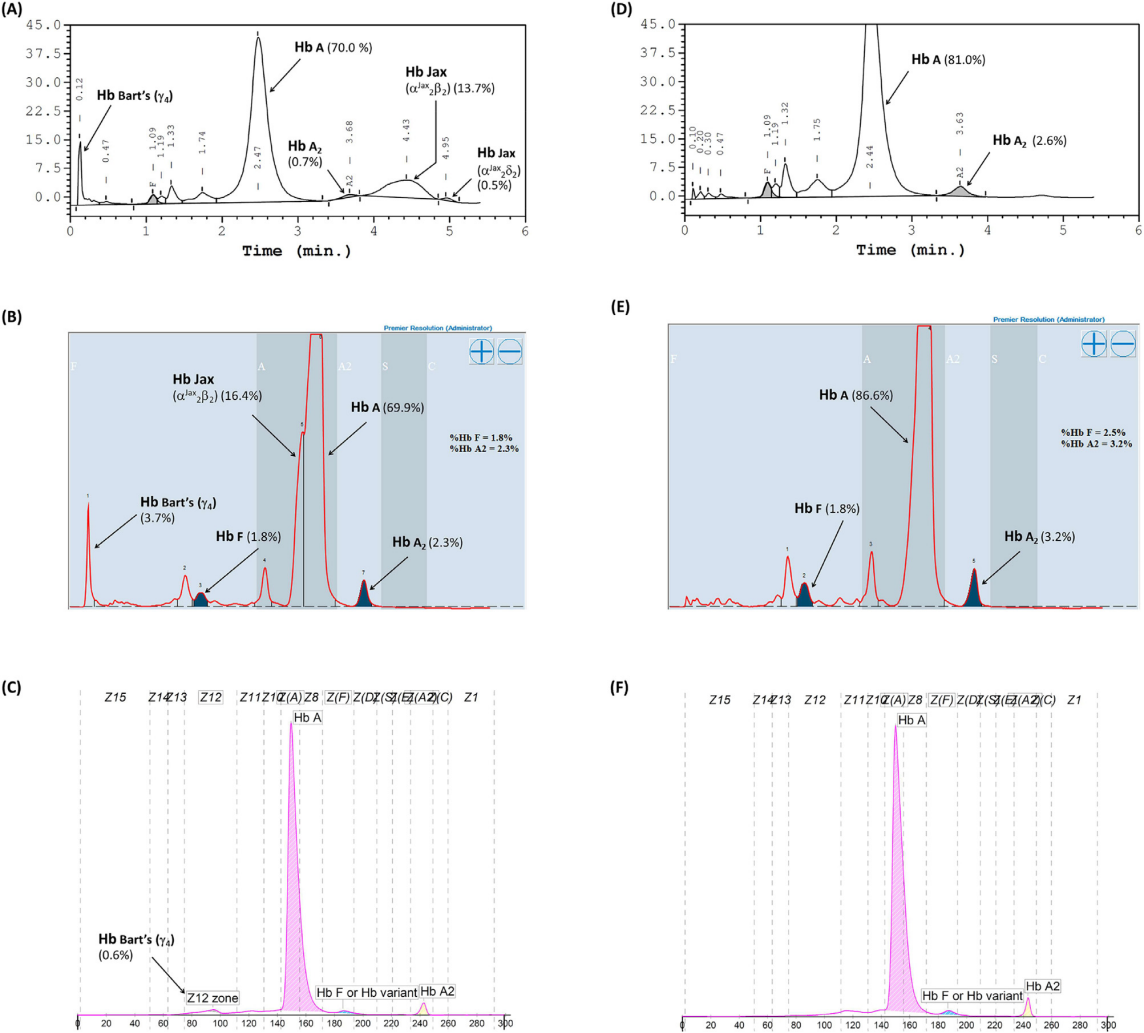
Hemoglobin analysis of the patient carrying Hb Jax co-inherited with α^0^-thalassemia (A-C) and her daughter carrying heterozygous Hb Jax (D-F). Panels A and D were obtained from analysis using the HPLC-variant II system, panels B and E from the HPLC-premier resolution system, and panels C and F from capillary electrophoresis.

Hb analysis using the Variant-II HPLC system disclosed two anomalous Hb peaks eluted after Hb A_2_. One of these peaks, eluting at a specific retention time of 4.43 mins, accounted for 13.7% of the total Hb, while the other peak, at 4.95 mins, constituted 0.5% (Figure 1A). Additionally, Hb Bart's, indicative of excessive free γ-globin chain due to α-globin chain synthesis exhibiting a marked decrease, was also distinctly seen on the chromatogram. A comparable analysis using the Premier Resolution-HPLC system, a novel cation exchange chromatography capable of discerning and quantifying Hb A_2_ even in the presence of Hb E, demonstrated Hb Bart's at approximately 3.7% of the total Hb. Furthermore, an anomalous Hb peak preceding Hb A was evident, albeit not entirely separated from Hb A, with a specific retention time of 4.249 mins, representing approximately 16.4% of the total Hb (Figure 1B). Conversely, capillary electrophoresis (CE) Hb analysis illustrated fractions of Hb Bart's, Hb A, and Hb A_2_, but no anomalous Hb peak (Figure 1C). The anomalous Hb peak was absent in the Hb analyses of the daughter, performed using both HPLC and CE systems (Figure 1D-1F). A comprehensive overview of the Hb analysis profiles and levels of individual separated Hb fractions for the patient and her daughter is presented in Table 2.

**Table 2:** Hemoglobin analysis profile and level of separated hemoglobin fractions of patient and her daughter carrying Hb Jax.

Hb analysis	Patient	Daughter
HPLC—Hb Profile^a^	A_2_ABart's and Hb Jax	A_2_A
Hb A (%)	70.0	81.0
Hb A_2_ (%)	0.7	2.6
Hb Jax (%)	13.7	0.0
Hb A_2_-Jax (%)	0.5	0.0
Hb F (%)	1.4	2.1
HPLC—Hb Profile^b^	A_2_ABart's and Hb Jax	A_2_A
Hb A (%)	69.9	86.6
Hb A_2_ (%)	2.3	3.2
Hb Jax (%)	16.4	0.0
Hb F (%)	1.8	2.5
Hb Bart's (%)	3.7	0.0
CE-Hb Profile^c^	A_2_ABart's	A_2_A
Hb A (%)	96.2	95.8
Hb A_2_ (%)	2.3	2.8
Hb Bart's (%)	0.6	0.0

HB, Hemoglobin; HPLC, High-performance liquid chromatography; CE, Capillary electrophoresis.

a Determined using HPLC (VARIANT II).

b Determined using HPLC (Premier resolution).

c Determined using capillary electrophoresis (MINICAP).

### Detection of α-globin gene abnormality

**Figure 2:**
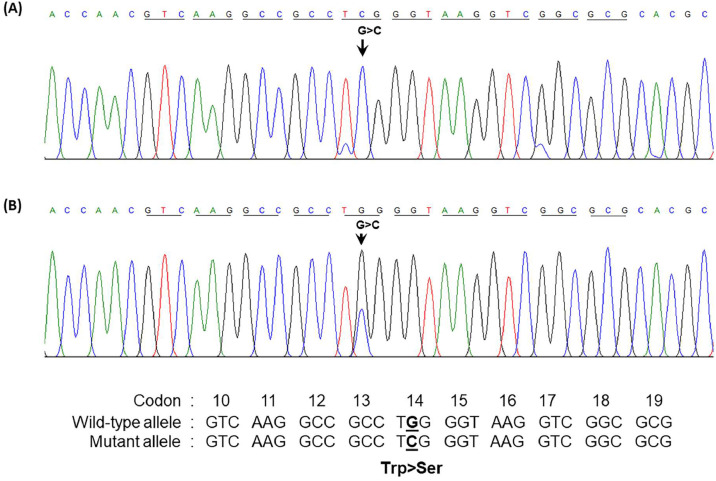
Forward directional nucleotide sequence analysis of the α_2_-globin gene of the patient (A) and her daughter (B) Downward arrow indicates the G to C transition at codon 14. (A) Co-inheritance of Hb Jax with α^0^-thalassemia (–^SEA^/α^14G>C^α) is shown, with only C at that position being identified. (B) heterozygous Hb Jax (α^14G>C^α/αα) showing G/C at this position. Trp, tryptophan; Ser, serine.

The presence of a low percentage of anomalous Hb coupled with Hb Bart's caused suspicion regarding a potential α-globin gene abnormality in the patient. Consequently, sequencing of the α-globin gene was undertaken. The DNA sequencing results unveiled a T*G*G>T*C*G transition at codon 14 of the α_2_-globin gene in the patient and her daughter. A homozygous alteration of T*G*G>T*C*G was observed in the patient (Figure 2A), and her daughter exhibited the change in a heterozygous state (Figure 2B). This transition led to the conversion of tryptophan to serine, which is responsible for Hb Jax [HBA2:c.44G>C]. This conversion was recently noted in the HbVar database (Hoyer JD, personal communication) and was previously undescribed in any Asia population. Subsequent DNA analysis of the α-globin gene showed the presence of a Southeast Asian (SEA) α^0^-thalassemia deletion in the patient. Thus, she was established as a compound heterozygote, bearing the combination of Hb Jax with α^0^-thalassemia. Notably, this deletion was absent in her daughter. Consequently, the patient's genotype was confirmed as –^SEA^/α^Jax^α, whereas her daughter's genotype was established as αα/α^Jax^α.

### Hemoglobin stability testing and inclusion bodies

**Figure 3:**
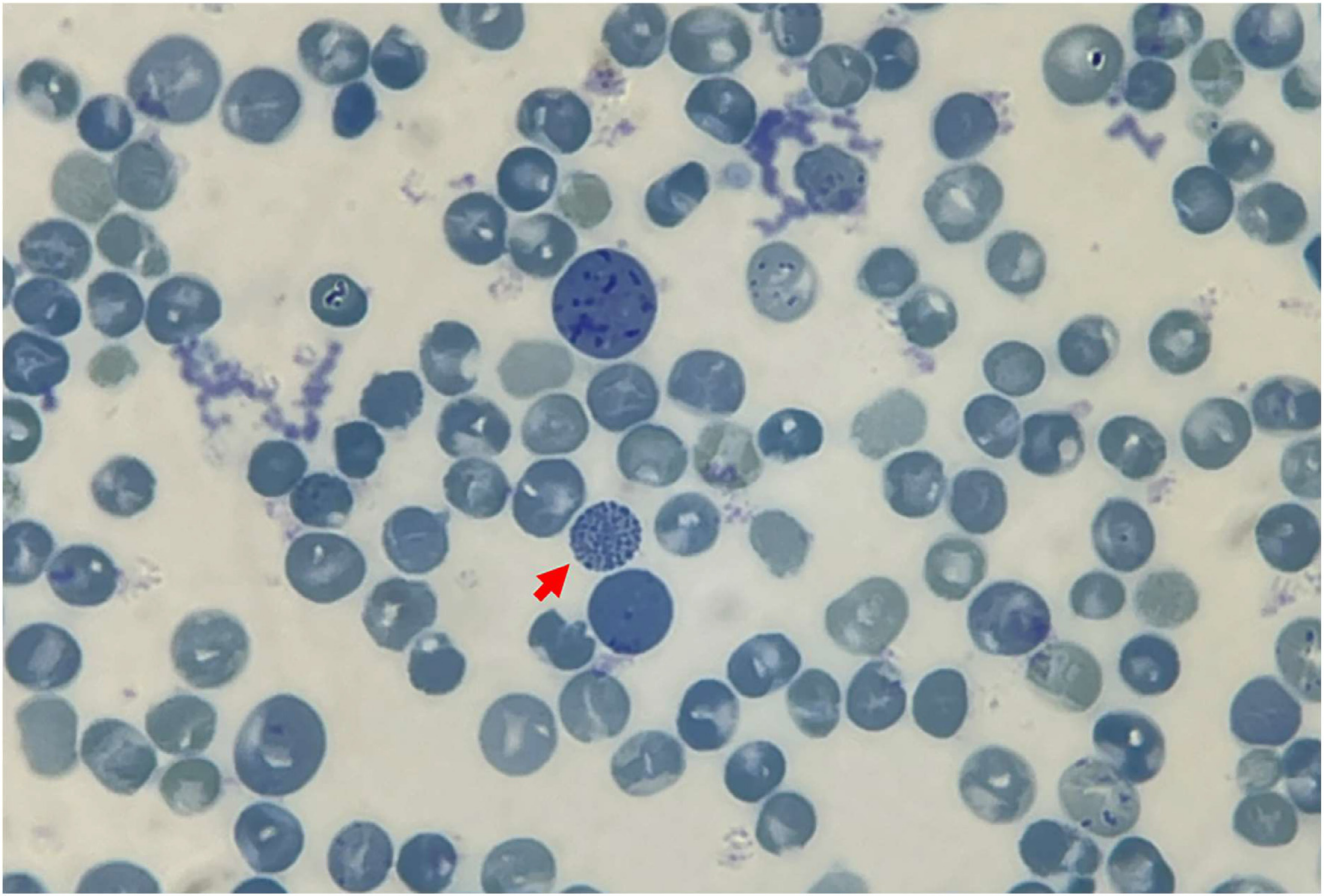
Formation of red blood cell inclusion bodies induced after incubating red blood cells with 1% brilliant cresyl blue for 3 h. Blue golf-ball-like formations (red arrows) are observed in the patient.

Hb stability testing revealed the formation of a minor flocculent precipitate following the extension of a standard isopropanol stability test to a 40-min incubation period. Positive results emerged from the test for inclusion bodies, with golf ball-like inclusions discernible in <1% of total peripheral RBCs (Figure 3).

### Molecular model and pathogenicity prediction

**Figure 4:**
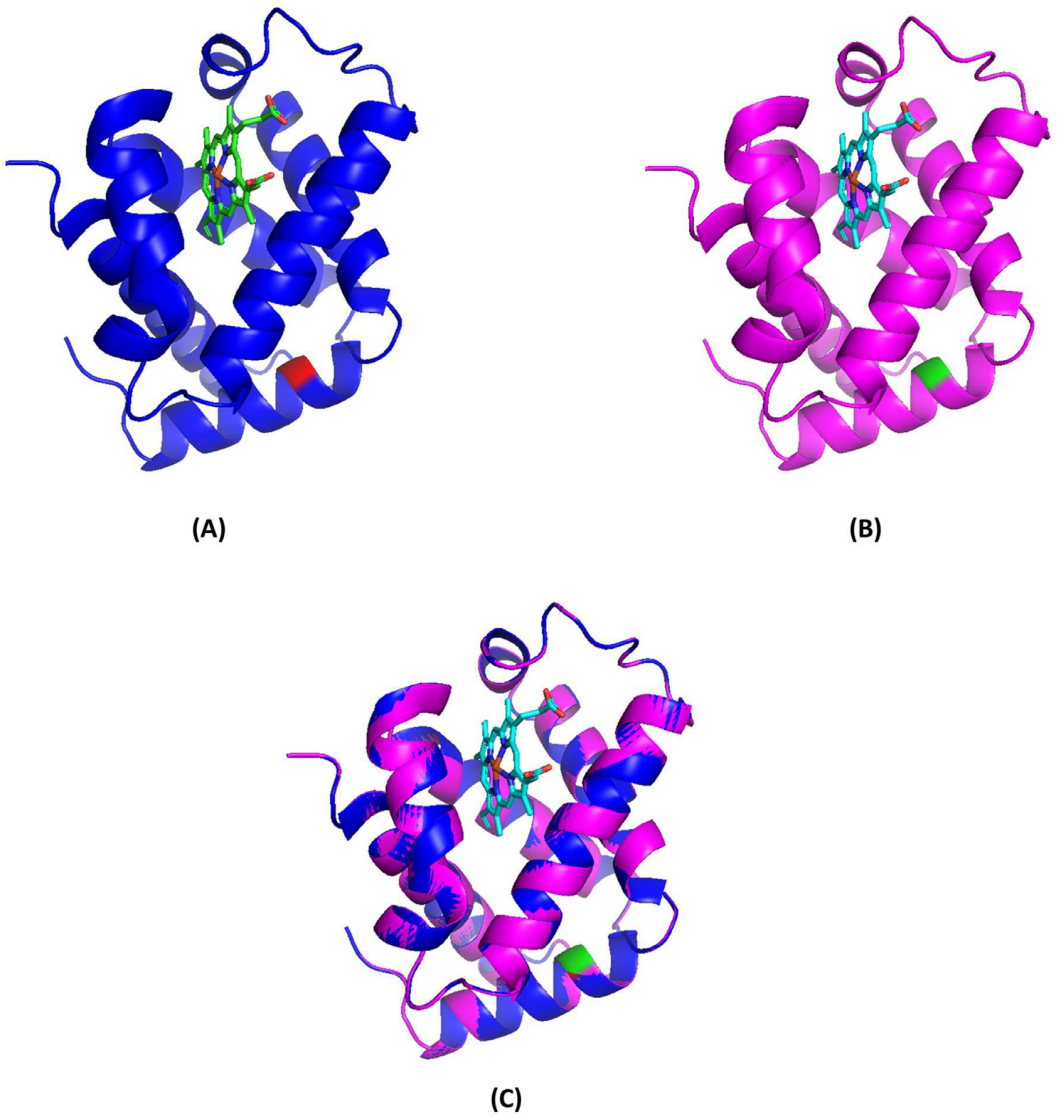
Molecular model of deoxyhemoglobin demonstrating the α-globin chain in a ribbon structure. (A) Normal α-globin protein, with Trp14(A12), indicated in red, and the heme group is shown as sticks. (B) Homology model of a mutant α-globin chain representing Hb Jax, with Ser14(A12) and the heme group shown in green and sticks. (C) Superposition of the model (pink) onto the template (blue).

The molecular model was constructed using the SWISS-MODEL, and the resulting depiction is illustrated in Figure 4 using PyMOL software. This analysis demonstrated that the structure of the mutant tetramer closely resembles that of the native Hb molecule, suggesting that the replacement of Trp by Ser at codon 14 (predicted to be situated within the α-helix structure) did not significantly alter the tertiary structure when compared to the non-mutant tetramer. Furthermore, the pathogenicity prediction for Hb Jax, as per SIFT and PolyPhen-2 models, indicated a deleterious effect, with a SIFT score of 0.00 and a PolyPhen-2 score of 0.991, classifying it as probably damaging.

### Confirmation of mutated Hb variant via allele-specific polymerase chain reaction

**Figure 5:**
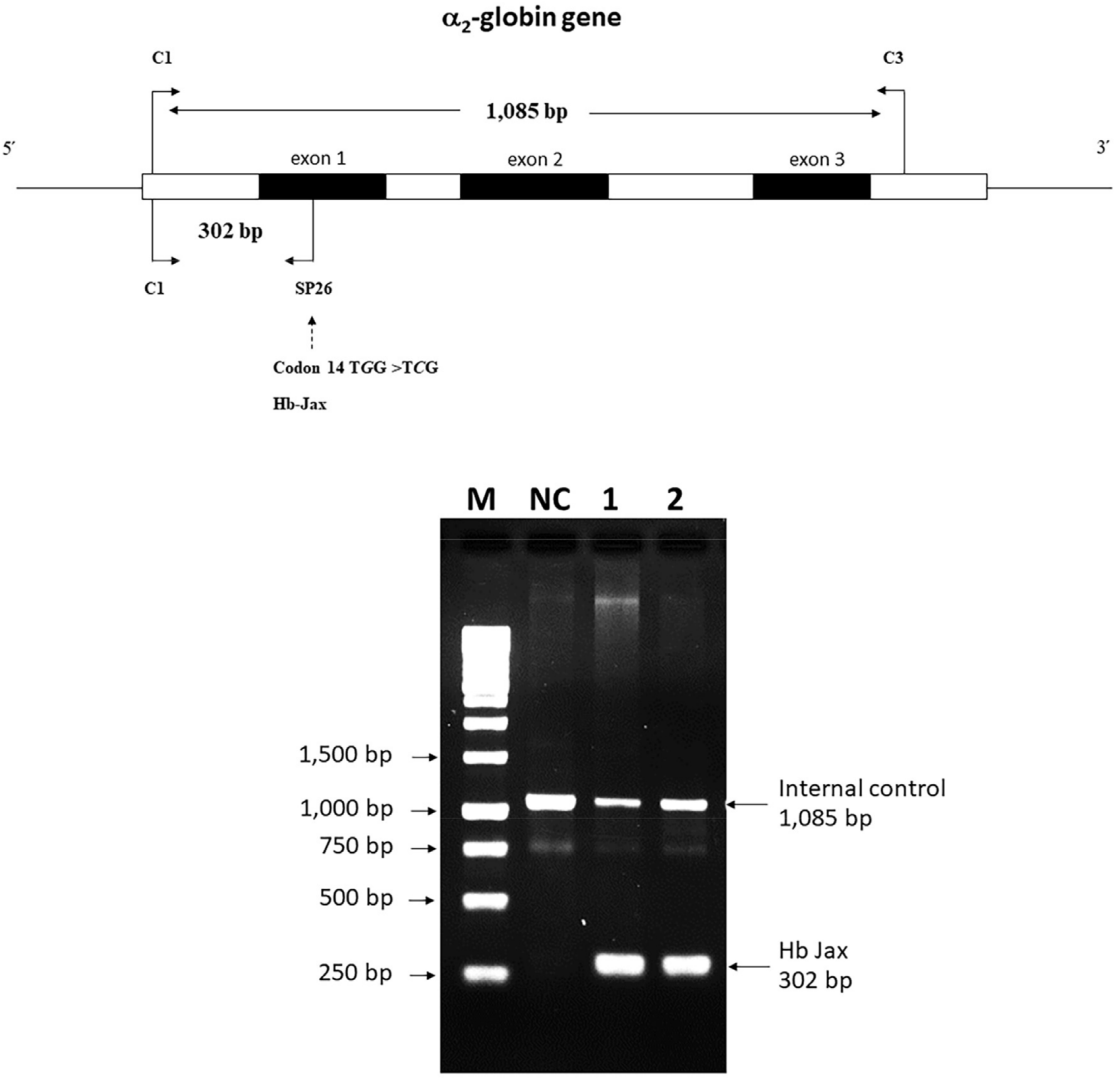
Detection of the Hb Jax mutation using 1.5% agarose gel electrophoresis of amplified fragments by allele-specific polymerase chain reaction (PCR). M represents the 250-base pairs (bp) leader DNA markers. The amplification product specific to the normal allele is seen observed at 1085 bp and the mutant allele at 302 bp. Lanes NC represent DNA negative control. Lanes 1 and 2 correspond to the patient and her daughter, respectively.

The presence of the mutated Hb variant, identified by DNA sequencing, was corroborated using a novel allele-specific PCR technique. In both the patient and her daughter, a distinct 302-bp amplified fragment specific to the Hb Jax allele was observable, contrasting the absence of this fragment in a normal individual. Furthermore, the 1085-bp fragment specific to the normal allele was also detected (Figure 5). This outcome underscores the efficacy of the newly developed allele-specific PCR technique for the accurate and rapid diagnosis of Hb Jax.

### Analysis of the α-globin gene haplotype

The α-globin gene haplotype analysis revealed [+ - *S* + - + -] from the Hb Jax allele.

## Discussion

This case identified and presented evidence supporting the pathogenicity of a rare α-thalassemic Hb variant, namely Hb Jax [HBA2:c.44G>C] in a Thai patient with unexplained chronic anemia but without any history of hepatosplenomegaly. This variant is defined by a transition from T*G*G>T*C*G at codon 14 of the α_2_-globin gene, leading to the substitution of tryptophan with serine. The mobility behavior of Hb Jax (α^Jax^_2_β^A^_2_) in HPLC is expectedly distinct from that of Hb A. Notably, Hb Jax elutes more slowly than Hb A. This divergence is critical because it ensures the complete separation of Hb Jax from Hb A using the HPLC-Variant-II system. This capability permits the accurate estimation of Hb Jax levels. Furthermore, the derivative Hb Jax (Hb A_2_-Jax), resulting from the assembly of two α^Jax^ and two δ chains into tetramers, is also entirely separated from Hb A_2_ and quantifiable via this system. Conversely, when subjected to analysis by the Premier-Resolution HPLC system, Hb Jax demonstrates a marginally faster elution than Hb A. Consequently, this system only partially separates Hb Jax from Hb A, making it challenging to accurately estimate their respective quantities when compared with the HPLC variant-II system.

Additionally, an eluted fraction of Hb A_2_-Jax was indiscernible, suggesting the possibility of co-elution with Hb A_2_. While the chromatograms of the patient displayed excellent resolution between normal Hb and the mutant Hb, the chromatogram of the heterozygote did not exhibit any noticeable peak, indicating that co-presence with other α-thalassemia might be necessary for visibility via chromatographic techniques. One plausible explanation for this divergence lies in the substantial formation of the Hb Jax tetramer in conjunction with α^0^-thalassemia. In this scenario, the limitation of α-globin production leads to a more pronounced expression of the α^Jax^ chain, owing to an affected mutation on the α_2_-globin gene, compared with the α^A^ chain derived from the α_1_-globin gene. This accelerated expression facilitates the rapid incorporation of excess unbound β-chains into the newly synthesized α^Jax^_2_β^A^_2_ tetramer. The use of the CE technique for Hb analysis resulted in indiscernible anomalous Hb peaks in both heterozygotes and compound heterozygotes with α^0^-thalassemia. This was suggestive of Hb Jax and its derivative co-migrating with Hb A and Hb A_2_. This phenomenon might be attributed to the substitution of the amino acid serine, an uncharged amino acid similar to tryptophan. Consequently, the net charge within the Hb tetramers remains unaltered. It is evident that reliance solely on the CE technique for Hb separation can lead to the misidentification of Hb Jax and other variants with physicochemical similarities.

The hydrophobic tryptophan residue, occupying position 14, is situated within a helical A segment of the α-chain. This location rests on the internal surface of the tetramer and does not engage in intersubunit contacts, including α1β1 or α1β2, nor does it interact directly with the heme group.^5^^,^^23^^,^^24^ Despite its apparent seclusion, this region holds a pivotal role as a highly conserved hydrophobic residue.^24^, ^25^, ^26^ The substitution of this vital position in Hb Jax with a polar serine residue, commonly found on the molecule's external surface, introduces a heightened degree of hydrophilicity to this region. Consequently, a noticeable transformation occurs in the physicochemical attributes of the Hb Jax tetramer, ultimately influencing its molecular stability. Bioinformatic predictions support this observation, proposing that Hb Jax's physiochemical disparities between tryptophan and serine render it potentially damaging and deleterious.^27^ Furthermore, a reduced molecular stability of this variant was successfully demonstrated using isopropanol stability testing. This is evidenced by the gradual increase in flocculent precipitate over time, indicating the minor instability of Hb Jax.

An intriguing feature of Hb Jax is the absence of detectable Hb H (β_4_ tetramers) despite employing CE and HPLC techniques for Hb separation. This is remarkable because the patient possesses only two α-globin genes, one of which carries the T*G*G>T*C*G mutation (α^Jax^). However, her phenotype and elevated reticulocyte count are notably similar to individuals with Hb H.^28^, ^29^, ^30^ Based on this observation, we infer that the increased imbalance in co-inheritance with the deletion defects did not lead to a substantial overproduction of β-globin chains, thus preventing the significant formation of β_4_ tetramers in peripheral RBCs. This suggests that the Hb Jax mutation only marginally impacts the synthesis of the α^Jax^ chain. However, in Hb Bart's, the accumulation of excess γ-globin chains produced in this patient was consistently observed through CE and HPLC techniques. Notably, the quantified percentage of Hb Jax using HPLC in the patient is less than 25% of the total circulating Hb, which is lower than the expression level of the functional α_2_ globin gene.^31^ Chemical evidence for supporting the variant's instability was established by the positive results from the test for inclusion bodies with brilliant cresyl blue and the positive outcome of the isopropanol tests for unstable Hb. These findings align with the low levels of the variant, leading us to conclude that the significant reduction in Hb Jax levels in this context results from denaturation and proteolytic degradation of Hb Jax tetramers rather than a reduction in synthesis due to mRNA instability. Consequently, we consider Hb Jax to be a slightly unstable Hb variant equivalent to α^+^-thalassemia.

The phenotypic expression in the heterozygosity of Hb Jax observed in the daughter of the patient is greatly compatible with individuals displaying a typical α^+^-thalassemia trait. This combination presents iron deficiency and more abnormalities in RBC parameters compared with those observed in individuals with the α^+^-thalassemia trait alone. The exact phenotype influenced by the mutant gene will be elucidated after their ferritin levels are improved.^32^ Although we have not provided information about another individual with pure heterozygous Hb Jax, it is reasonable to posit that the phenotypic expression of a person with a pure heterozygote of this variant would be highly similar to that of an individual with the α^+^-thalassemia trait. The severe clinical phenotype observed in the patient can be attributed directly to the co-inheritance of two α-globin gene deletions in *trans*, as there is no evidence suggesting the presence of iron deficiency or other causes of microcytic anemia in the patient. The frequency of Hb Jax in the Thai population remains unknown through routine Hb separation alone due to the technological limitations for Hb analysis that fails to virtually detect the Hb Jax fraction in heterozygotes. DNA level analysis is required to accurately determine the prevalence of this variant, particularly in a population with a high prevalence of α-thalassemia determinants. Such investigations would prove valuable for genetic counseling. Additionally, our patient had moderate anemia, marked microcytosis, and hypochromia. The analysis of common α^+^-thalassemia genes yielded negative results, underscoring the necessity to investigate this mutation in the α-globin gene, which may interact with other forms of thalassemia or hemoglobinopathies.

A mutation at codon 14 of α_2_-globin has been reported in Hb Evanston [HBA2:c.[43T>*A* (HBA1) or 43T>C (HBA1)].^33^ The relatively unstable Hb Evanston is associated with defective tetramer stability and globin chain synthesis as the α^Evanston^ gene exists on a chromosome *cis* to a -α^3.7^gene. When linked with thalassemia, this variant contributes to an α-thalassemia-like phenotype, potentially leading to Hb H when co-inherited with another α-thalassemia defect,^34^ as observed in our patient. The Hb Evanston is virtually undetectable in heterozygous individuals using CE and HPLC techniques.^35^ In contrast, its combination with an additional α-thalassemia deletion gene allows for more readily detectable quantities of Hb Evanston, approximately 1–2% in association with the leftward deletion in *trans* and 6–10% in association with two α-globin gene deletions.^34^^,^^35^ The findings in the Hb Evanston and Hb Jax emphasize that the Hb variant tetramer resulting from the affected α_2_-globin gene mutation on codon 14 is relatively unstable, generating the α-thalassemia-like phenotype.

Analyzing the α-globin haplotype provides a better understanding of the origins and spread of this variant within the population. The α-haplotype associated with this variant is similar to that found in Hb Prato, documented in a Thai patient,^15^ indicating a shared evolutionary origin stemming from this ancestry.

## Conclusions

Hb Jax is a rarely identified α-globin variant resulting from the substitution of tryptophan with serine at codon 14 of the α_2_-globin gene. It displays susceptibility to tetramer instability. In a heterozygous state, it manifests as marginally abnormal hematologic findings similar to α^+^-thalassemia. However, when combined with an α^0^-thalassemia gene in *trans*, it assumes a clinically significant role, resembling an Hb H-like phenotype. Notably, the mutation remains cryptic on a wild-type allele but becomes visible through chromatographic techniques when associated with other α-thalassemic defects. These findings represent the first description of this variant in published literature. The developed allele-specific PCR approach improved the accuracy and speed of diagnosing this variant, with potential applications for comprehensively studying its prevalence in regions with high incidence of common α-thalassemia determinants.

## Author contributions

SP performed study concept and design, development of methodology and writing, review and revision of the paper; KJ, PK, PN were involved in analysis and interpretation of data; SS was involved in analysis and interpretation of data, and statistical analysis. All authors read and approved the final paper.

## Funding

This work was supported by the Thailand Science Research and Innovation Fund and the University of Phayao, Thailand (Grant number FF65-RIM135).

## Availability of data and materials

The data presented in this study are available upon request from the corresponding author.

## Conflicts of interest

The authors declare that they have no conflicts of interest to declare.
